# The Role of Reactive Oxygen Species in Modulating the *Caenorhabditis elegans* Immune Response

**DOI:** 10.1371/journal.ppat.1005923

**Published:** 2016-11-10

**Authors:** Katie C. McCallum, Danielle A. Garsin

**Affiliations:** Department of Microbiology and Molecular Genetics and The UT Center for Antimicrobial Resistance and Microbial Genomics, McGovern Medical School, The University of Texas Health Science Center at Houston Houston, Texas, United States of America; Tufts Univ School of Medicine, UNITED STATES

## Production of reactive oxygen species can positively contribute to immune responses

Reactive oxygen species (ROS) can elicit extreme biological damage by modifying DNA, proteins, and lipids. However, ROS also play diverse and beneficial roles, including involvement in the innate immune response. Mechanisms by which ROS affect innate immunity include direct killing of bacterial and fungal pathogens. The classic example is the destruction of microbes that occurs in the phagolysosome of innate immune cells when superoxide, produced by NOX2, is converted into the potent cytotoxic molecule hypochlorous acid by myeloperoxidase [1]. ROS can additionally play roles in innate immune signaling and in the formation of protective barriers [2].

While generated endogenously as a byproduct of aerobic respiration, ROS production must be tightly regulated in a spatial and temporal manner if it is to be efficiently utilized in host physiology. However, the main function of certain enzymes, such as the NADPH oxidase/dual oxidase (NOX/DUOX) family, is to produce ROS for specific purposes. Electrons from NADPH are utilized by these enzymes to catalyze the reduction of molecular oxygen, usually into superoxide anion. DUOXs are a subgroup of the NOX family that have an additional peroxidase homology domain and produce hydrogen peroxide. Intriguingly, however, human DUOXs are reported to lack peroxidase activity and often pair with peroxidase enzyme partners [3,4].

The mammalian NOX family encodes five NOX (Nox1–5) and two DUOX (Duox1–2) enzymes [4]. While it is clear that these enzymes play important physiological roles in humans, the presence of seven NOX family enzymes complicates the study of their individual roles and importance. Over the last two decades, the tiny roundworm *Caenorhabditis elegans* has emerged as a sophisticated model organism to study aspects of innate immunity, including its utilization of a single DUOX, called Ce-Duox1/BLI-3, to generate ROS during infection [5,6]. Therefore, the worm represents a unique opportunity to study, in isolation, the contribution of a single DUOX in the ROS-dependent elimination of pathogens in a natural, whole-organism context.

## *C*. *elegans* produces ROS via Ce-Duox1/BLI-3 during the immune response

The *C*. *elegans* genome encodes two NOX family enzymes, the DUOXs Ce-Duox1/BLI-3 and Ce-Duox2 [7]. However, only BLI-3 has characterized functional roles. Specifically, BLI-3 is required for proper development of the cuticle and is expressed in the hypodermis, pharynx, and intestine [7,8]. Partial loss of *bli-3* results in a severe blistering of the cuticle, while complete loss results in a nonviable animal [7,9]. Independent of the cuticle blistering, partial loss of *bli-3* has also been associated with pathogen susceptibility [5].

In response to infection by both bacterial and fungal pathogens, BLI-3-generated ROS play a role in both intestinal and epidermal immunity [5,10,11]. The intestinal lumen of the worm can be colonized by pathogens such as *Enterococcus faecalis* and *Candida albicans*, while the epidermis is subject to infection and wounding by other pathogens, such as the fungi *Drechmeria coniospora* [12–14]. The BLI-3-dependent production of ROS is critical for worm survival, and reducing the level of expression of *bli-3* significantly impairs its ability to survive infection with these pathogens [5,8,10]. During intestinal infection with *E*. *faecalis*, it has been demonstrated that the NADPH oxidase domain of BLI-3 is critical for this protective function; the peroxidase domain does not appear to have a role [5,8]. While it is known that BLI-3 is not regulated at the mRNA or protein level, the infection-dependent signals and corresponding regulatory components that control the stimulation of BLI-3 during intestinal-specific infection remain unclear [8,15], though there is some evidence that bacterial-derived uracil modulates the process [16]. In the case of epidermal infection by *D*. *coniospora*, the IP3-ITR-1Ca^2+^ pathway stimulates BLI-3 to release ROS, ultimately activating a protective immune response through Ste20-like kinase/CST-1-controlled activation of DAF-16 [10].

## Possible mechanisms by which ROS produced by BLI-3 are protective

Recall that DUOXs possess a peroxidase homology domain in addition to an NADPH oxidase domain. Unlike the mammalian DUOXs, BLI-3 exhibits low levels of peroxidase activity, which is important for worm cuticle biogenesis [7,17]. Moreover, similar to the functionally cooperative relationship between NOX2 and myeloperoxidase, cuticle biogenesis relies on an association between BLI-3 and a *C*. *elegans* peroxidase, MLT-7 [18].

Interestingly, however, the peroxidase activity of BLI-3 is not required for its role in the innate immune response. During bacterial infection, two strains harboring independent point mutations in the peroxidase domain of *bli-3* produced wild-type levels of ROS and did not exhibit the reduced survival characteristic of reduced *bli-3* levels [5,8]. Current evidence suggests that BLI-3 might rely on peroxidase partners during infection, the best characterized being SKPO-1. *skpo-1* mutants exhibit reduced survival during pathogen challenge. Additionally, significantly higher levels of hydrogen peroxide are produced in *skpo-1* mutants in a *bli-3*-dependent manner, consistent with SKPO-1 utilizing hydrogen peroxide produced by BLI-3. SKPO-1 and BLI-3 colocalize to the hypodermis, raising the possibility that their apparent functional interaction may involve a direct interaction [19]. A major unanswered question is, what is SKPO-1 utilizing hydrogen peroxide for during infection? One possibility is the generation of more potent antimicrobials, similar to the role of some mammalian peroxidases that generate hypochlorous acid or hypothiocyanite [1,20]. Second, SKPO-1 may utilize BLI-3-generated ROS to strengthen the cuticle of the worm, by cross-linking collagen proteins, similar to the role MLT-7 and the peroxidase domain of BLI-3 play during cuticle development [18].

## Alternative sources of ROS are protective during infection

In addition to NOX family enzymes producing ROS, increasing evidence suggests that other host-derived sources of ROS are important components of innate immunity. Both mouse and zebrafish models of infection demonstrated that elevation of mitochondrial ROS (mtROS) is a critical component of innate immunity, as loss of this capability results in sensitivity to pathogenic bacteria [21,22]. Recently, mtROS have been shown to play important roles in *C*. *elegans* immunity. Microarray analysis indicates that increased expression of several innate immune genes occurs as a result of genetic inhibition of mitochondrial respiration, which is known to result in elevated ROS levels. Concordantly, the ROS release stimulated upon inhibition of mitochondrial respiration promotes worm resistance to several bacterial pathogens, including *E*. *faecalis*, *Pseudomonas aeruginosa*, and pathogenic *Escherichia coli*. While little is known regarding the downstream effectors of mtROS, their survival-enhancing effect depends on the hypoxia-inducible factor 1 (HIF-1) transcription factor and 5′ adenosine monophosphate-activated protein kinase (AMPK) cellular energy sensor [23]. In addition to an importance in immunity, mtROS have also recently been shown to play an important role in epidermal wound repair, which can occur during pathogen infection [24]. Elevated calcium levels signal actin accumulation to occur at wound sites by an unclear mechanism. Using both pharmacological and genetic methodologies, Xu et al. demonstrated that a wounding-dependent increase in calcium stimulates mtROS release, facilitating the oxidation, and subsequent inhibition, of RHO-1 guanosine-5′-triphosphatase (GTPase,) a negative regulator of actin ring closure. Therefore, the mtROS-dependent oxidation of this small GTPase ultimately promotes actin-based wound repair [25]. Given that wounding can result from pathogen infection, it is possible that, in addition to HIF-1/AMPK-dependent resistance to pathogens, the worm innate immune response utilizes mtROS to repair the damage elicited by invading pathogens.

## ROS are signaling molecules that induce protection during infection

There is precedence for ROS being utilized as signaling molecules during the immune response. For instance, hydrogen peroxide acts as a long-range signaling molecule important for attracting leukocytes to wound sites in zebrafish [26]. Additionally, ROS are signaling mediators important for the activation of nuclear factor kappa B (NF-κB) and p38 mitogen-activated protein kinase (MAPK), crucial regulatory components of many mammalian processes, including immunity [27,28]. While ROS signaling has been implicated in playing important roles in several pathways that influence aging and metabolism, the role of ROS-dependent signaling on innate immune response activation is understudied in the worm [29]. There is mounting evidence, however, that ROS play a signaling role in the activation of protective responses.

An unfortunate consequence of utilizing ROS as an antimicrobial is the collateral damage to the host that occurs if redox homeostasis is not properly maintained [30]. Therefore, during infection, the worm employs transcription factors, such as SKN-1 and DAF-16, to increase the expression of oxidative stress enzymes [31–33]. Initial studies did not observe a role for the *C*. *elegans* major oxidative stress response transcription factor SKN-1 during infection [34,35], but further work, with additional controls, carried out by two independent laboratories discovered SKN-1 to be protective [32,33]. During infection, SKN-1 activation increases the expression of antioxidants in a BLI-3-dependent manner enhancing survival. Moreover, the p38 MAPK pathway is responsible for relaying the signal to activate SKN-1 during infection [32,33]. Recently, a neuronally encoded thioredoxin, TRX-1, was demonstrated to impact SKN-1 subcellular localization, suggesting that this transcription factor may be cell nonautonomously regulated [36]. In this manner, SKN-1 maintains host redox homeostasis during infection. The increased sensitivity of *skn-1* mutants, and increased resistance of strains that overexpress *skn-1*, to pathogen infection underscores the physiological relevance of this regulation [33].

**Fig 1:**
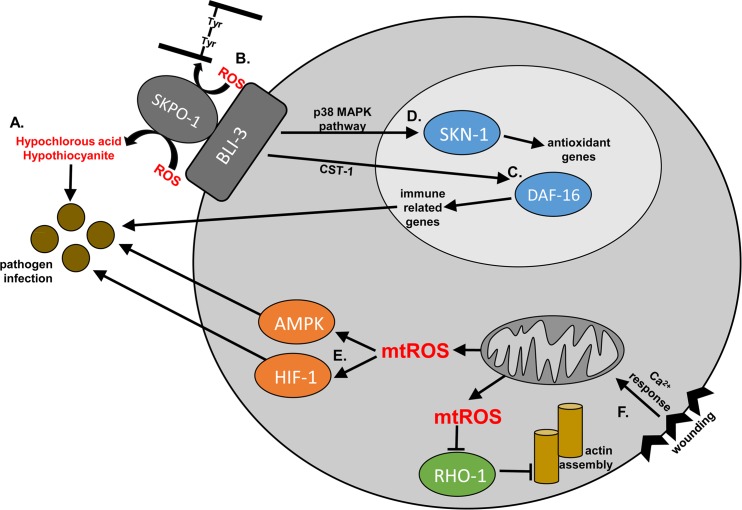
Possible mechanisms by which ROS are protective during infection. During pathogen infection, BLI-3-generated ROS may be utilized (A) by SKPO-1 to generate more potent antimicrobials (B) to maintain protective barriers; (C) to activate DAF-16, via oxidation of CST-1, to increase the expression of protective immune response genes; and/or (D) as a signaling molecule to activate SKN-1, via the p38 MAPK pathway, to maintain redox homeostasis during infection. Alternatively, pathogen infection stimulates the release of mitochondrial ROS (mtROS). These mtROS (E) facilitate HIF-1 and AMPK-dependent immunity. (F) Alternatively, during infection, wounding-dependent release of calcium causes mtROS release, relieving repression of protective actin assembly via oxidation of RHO-1.

Overall, recent findings support a role for ROS in the immune response of *C*. *elegans* by stimulating critical signaling pathways that elicit protection as summarized in Fig 1. ROS may also be used for generating potent antimicrobials and/or reinforcing barrier functions; definitive answers await future work.
